# Genetic diversity and population structure of *Rhipicephalus sanguineus* sensu lato across different regions of Colombia

**DOI:** 10.1186/s13071-021-04898-w

**Published:** 2021-08-23

**Authors:** Luisa Páez-Triana, Marina Muñoz, Giovanny Herrera, Darwin A. Moreno-Pérez, Gabriel A. Tafur-Gómez, Diego Montenegro, Manuel A. Patarroyo, Alberto Paniz-Mondolfi, Juan David Ramírez

**Affiliations:** 1 Centro de Investigaciones en Microbiología y Biotecnología-UR (CIMBIUR), Facultad de Ciencias Naturales, Universidad del Rosario, Bogotá, Colombia; 2 Universidad de Ciencias Aplicadas y Ambientales—U.D.C.A., Bogotá, 111166 Colombia; 3 Fundación Chilloa, Santa Marta, Colombia; 4 Molecular Biology and Immunology Department, Fundación Instituto de Inmunología de Colombia (FIDIC), Bogotá, Colombia; 5 Microbiology Department, Faculty of Medicine, Universidad Nacional de Colombia, Bogotá, D.C. Colombia; 6 Health Sciences Division, Main Campus, Universidad Santo Tomás, Bogotá, D.C. Colombia; 7 Icahn School of Medicine at Mount Sinai, New York, NY USA

**Keywords:** *Rhipicephalus sanguineus*, Genetic diversity, Phylogeny, Tick, Vector-borne diseases

## Abstract

**Background:**

There has been a long-standing debate over the taxonomic status of *Rhipicephalus sanguineus* sensu lato. Different studies worldwide have reported the occurrence of different well-defined lineages, in addition to *Rhipicephalus sanguineus* sensu stricto. To date, there are very few studies examining the diverse aspects of this tick in Colombia. We assessed the population structure and genetic diversity of *R*. *sanguineus* s.l. in eight departmental regions across Colombia.

**Methods:**

A total of 170 ticks were collected from dogs in different departments of Colombia. All specimens were morphologically compatible with *R*. *sanguineus* s.l. and subjected to genetic analysis. DNA sequences were obtained for the 12S rDNA, cytochrome oxidase I (*COI*) and internal transcribed spacer 2 (ITS2) markers. A concatenated set of all mitochondrial markers was also constructed. Next, maximum likelihood phylogenetic trees were constructed using the sequences generated herein and sequences available in GenBank. Finally, we assessed different summary statistics and analysed population structure and divergence with Fst and Dxy and demographic changes with Tajima's *D* and Fu and Li’s statistical tests.

**Results:**

Analysis of the 12S rDNA and *COI* revealed that all *R*. *sanguineus* s.l. specimens collected across different regions of Colombia clustered within the tropical lineage. Micro-geographical analyses showed that the tick population from Amazonas formed a distinct cluster separated from the other sequences, with moderate Fst and Dxy values. However, no signs of a robust population structure were found within the country. The results of Fu’s *F*_S_ tests, together with the haplotype networks and diversity values, signal a possible population expansion of this tick species in Colombia.

**Conclusions:**

Evidence provided herein supports the tropical lineage as the main circulating lineage in Colombia, exhibiting a general lack of genetic structure except for the Amazonas region.

**Graphical Abstract:**

**Figure:**
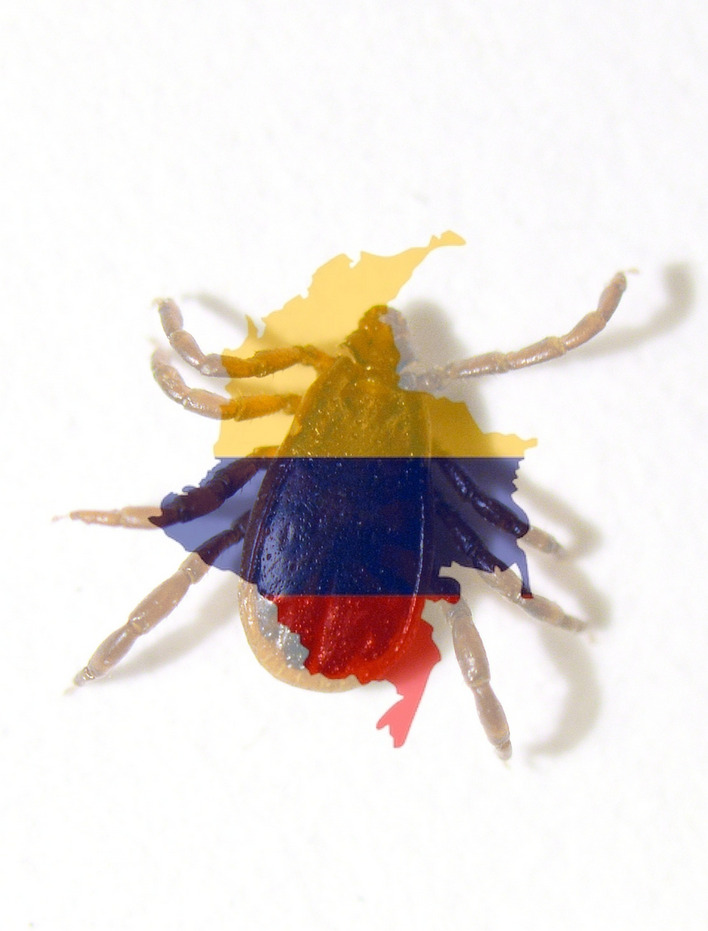

**Supplementary Information:**

The online version contains supplementary material available at 10.1186/s13071-021-04898-w.

## Background

Ixodid ticks are one of the most important pathogen vectors for humans and domestic animals worldwide [1]. Ticks of the genus *Rhipicephalus* belong to the Metastriata group, which includes species distributed across most continents, displaying the most abundant diversity of species, mostly in Africa [2, 3]. *Rhipicephalus sanguineus* sensu lato, also known as the brown dog tick, is probably the tick with the largest global distribution [4], primarily across tropical and subtropical realms. These arthropods are of great public health and economic importance given that they serve as efficient vectors for multiple pathogens [5–7], and considering their ability to infest almost all vertebrates [8]. In addition, because of their trioxenic nature, *Rhipicephalus* spp. ticks are known to cause high morbimortality in livestock leading to huge losses in meat and dairy production [9]. Indeed, *Rhipicephalus* spp. ticks have also been identified as vectors for the causative pathogens of spotted fever group rickettsioses, babesiosis, ehrlichiosis and hepatozoonosis among others [8, 10].

Based on its ancestral behaviour and shared morphological traits in common with other tick species, *R*. *sanguineus* s.l. is considered to be a complex of species [2, 6], including *R*. *sanguineus* sensu stricto described by Latreille in 1806. The taxonomic classification of *R*. *sanguineus* s.s. has long been a matter of debate [6, 7, 11], but recently solved a neotype designation with the molecular characterization and morphological re-descriptions of all parasitic stages of this tick species [12]. Descriptions have shown that this group of ticks exhibits a broad variability of morphological variations [7]. For example, in Africa, male populations may show wide variations in the size of their posterolateral grooves, density of scapular punctuation patterns and shape of adanal plates, among other morphological features [2]. Furthermore, studies have shown that diverse populations of *R*. *sanguineus* s.s., frequently identified using traditional taxonomic keys [2], are in fact genetically and/or biologically divergent species [13–18]. As a result, consensus has been reached to refer to this taxon as *R*. *sanguineus* s.l., unless it is assigned to reference sequences of *R*. *sanguineus* s.s. (see Nava et al. [12]).

Genetic studies interrogating mitochondrial genes such as 18S rDNA, cytochrome oxidase (*COI*) and 12S rDNA have demonstrated the existence of two major distinct lineages, whose distribution is tightly linked to geographical and climate patterns. The first is located between latitudes 25°N and 22°S and the second is found closer to the poles, above 25°N and below 22°S [13]. Studies from Latin America have also shown differences in distribution between the lineages, with the first clade geographically distributed between Mexico and Brazil, and the second clade settling towards the southern cone of the continent [14]. As a result, the terms “tropical” and “temperate” (= *R*. *sanguineus* s.s.) have been coined to define the presence of these two major lineages [14]. This notion was further supported by Nava et al. [15], who reported a similar pattern of occurrence, with the tropical lineage (represented by *R*. *sanguineus* s.l.) present in Paraguay and tropical regions of Argentina, and *R*. *sanguineus* s.s. found at more temperate localities in Chile, Uruguay and Argentina. In relation to vector competence, it is known that pathogen transmission varies in relation to lineages, with the tropical lineage exhibiting higher vector competence and increased transmissibility in contrast to *R*. *sanguineus* s.s. [19].

A more in-depth study by Dantas-Torres et al. [16] further confirmed the occurrence of two *R*. *sanguineus* s.l. lineages, using a combined morphological and genetic approach, and including samples from every continent. The study also shed light on the existence of three additional independent lineages (*Rhipicephalus* sp. I, *Rhipicephalus* sp. III and *Rhipicephalus* sp. IV) that could represent previously described or newly discovered species [16]. From a morphological perspective, high similarity was observed between species. However, the authors were still able to discriminate morphologically upon careful examination [16]. Interestingly, this study also included samples from Colombia, showing that most of the collected species at a regional scale fell into the tropical lineage [16], confirming previous findings by Moraes-Filho et al. [17]. Still, the presence of a haplotype previously identified within the temperate lineage [16] suggested the possibility of the occurrence and co-circulation of two or more lineages.

Parallel population studies aimed at establishing the systematic relationships within *R*. *sanguineus* s.l. and its potential biogeography showed a link between lineages and different bioclimatic variables, particularly temperature. Zemtsova and co-workers were the first to delineate the geographical distribution of *R*. *sanguineus* s.l. [20]. In their research, they identified that samples from the tropical lineage were found in regions where the average annual temperature was greater than 20 °C, whereas *R*. *sanguineus* s.s. was found at temperatures ranging between 10 and 20 °C [20]. However, despite this sharp geoclimatic demarcation, evidence suggests that biological plasticity and adaptation of *R*. *sanguineus* s.l. has allowed both lineages to co-occur in the same regions [20], such as has been reported in Brazil [12] and Argentina [20].

A number of studies have provided information about the biology and genetic diversity of *R*. *sanguineus* s.l. at a global scale. However, studies on the genetic variability of this tick in Colombia and across its diverse ecosystems are still scarce [16]. Also, phylogenetic relationships studies and estimates on genetic structure of *R*. *sanguineus* s.l. have yet not been conducted. In this study, we aimed to assess the current genetic diversity of *R*. *sanguineus* s.l. ticks across departments and various biogeographical regions including Amazonas, Andes, Caribbean, Orinoco and the Pacific.

## Methods

### Collection of samples

A total of 170 adult *R*. *sanguineus* s.l. ticks were collected from owned dogs, across eight different departments encompassing five out of the six main ecoregions in Colombia. Samples from the Amazon region included 59 ticks from the Amazonas Department (4°12′19″S, 69°55′58″W) and two from Putumayo (0°37′S, 77°16′W). Samples from the Andean region included eight ticks from Cundinamarca (4°36′N, 74°05′W). From the Caribbean region, four ticks from Bolívar (10°24′N, 75°30′W), 38 from La Guajira (11°33′N, 72°54′W) and 33 from Magdalena (11°14′N, 74°12′W) were included. Orinoco and Pacific region samples included 23 ticks from Meta (4°09′N, 73°38°W) and three from Valle del Cauca (3°25′N, 76°31′W), respectively. Additional information has been included in Additional file 1: Table S1.

Ticks were manually removed and stored in RNAlater [21] after collection. Based on life history, population density and ecological conditions, for practical purposes, the collection site was assumed to be the site of origin for all ticks. All ticks were initially identified based on morphological features [2, 12] and subsequently used for DNA extraction and DNA sequencing.

### DNA extraction, PCR and sequencing

DNA extraction was performed using a commercial kit (Quick-DNA Tissue/Insect Kit, Zymo Research, Irvine, CA, USA) following the manufacturer's recommendations, with the exception that bead-based disruption was replaced by manual disruption. DNA purity was assessed by measuring the absorbance at a 260/280 ratio using the NanoDrop system (Thermo Fisher Scientific), followed by sample storage at −20 °C until use.

Subsequently, the 12S rDNA (276 bp), *COI* (709 bp) and internal transcribed spacer 2 (ITS2; 1200–1600 bp) molecular markers were amplified by PCR in a Labnet thermal cycler (Labnet International) using previously reported primers sets (Additional file 2: Table S2) [13, 22–24]. In brief, each PCR reaction consisted of a mixture of GoTaq Green Master Mix (Promega, Madison, WI, USA) at 1× concentration, 1 µM of each primer, 4 µl of total DNA and 4.8 µl molecular biology grade water to complete a final volume of 20 µl. Amplification conditions for each marker are shown in Additional file 2: Table S3.

Amplicons were separated based on size in a 2% agarose gel stained with SYBR Safe and visualized in the MiniBIS Pro (DNR Bio Imaging Systems). Those that showed a single product were subsequently purified with ExoSAP (Thermo Fisher Scientific, Waltham, MA, USA) according to the manufacturer’s instructions and bidirectionally sequenced (with specific primers for each marker) using the Sanger method at Macrogen Inc. (Seoul, Korea). Geneious software [25] was used to verify and visually inspect the quality of the chromatograms and to perform de novo assembly to create consensus sequences per individual and genetic marker.

### Alignment and sequence analysis

The sequences obtained were aligned using the MUSCLE method [26] in UGENE [27] software for each genetic marker individually and in concatenation for mitochondrial genes (12S rDNA and *COI*), as previously reported for the identification of lineages traditionally accepted for *R*. *sanguineus* s.l. [16]. Analysis length was defined as follows: for 12S rDNA, 329 bp in 170 sequences obtained; for *COI*, 464 bp in 161 sequences; and for ITS2, 920 bp in 154 sequences. Concatenation of mitochondrial markers was carried out in alphabetical order, obtaining 146 sequences 790 bp in length.

In order to better represent the diversity for each marker, haplotypes were depicted by geographical regions, using DnaSP v6 software [28]. Haplotypes are interpreted here following NIH definitions as a combination of alleles in a defined region of the genome, involving single or multiple genes—as in the concatenated set—that tend to not recombine amongst different polymorphisms and are inherited together [29]. From these haplotypes, a second alignment was carried out by the above-described method. A third alignment including sequences available in GenBank was performed using publicly available sequences deposited as "*R*. *sanguineus*”, which included information regarding its geographical origin. A total of 42 sequences for the 12S rDNA marker, 66 for *COI* and 54 for ITS2 were included.

### Phylogenetic analyses

IQ-TREE version 1.6.12 [30] was used for maximum likelihood phylogenetic analysis. Trees were constructed for each individual genetic marker and the concatenated set, and later visualized by ITOL [31]. The best replacement model was chosen by jModelTest [32]. The support of the branches was carried out by means of 10,000 repetitions for ultrafast bootstrap approximation (UFBoot) [33] and with 10,000 repetitions for SH-like approximate likelihood test (SH-aLRT). Additionally, UFBoot was optimized by a hill-climbing nearest-neighbour interchange (NNI).

Finally, in order to observe the relationships between the sequences obtained for Colombia, haplotype networks were constructed by means of the TSC method in PopART (Population Analysis with Reticulate Trees) software [34]. Phylogenetic networks were constructed using the neighbour-joining (NJ) method in SplitsTree5 [35] to observe evolutionary histories of the phylogenies and possible cross-linking events.

### Genetic diversity and population structure

Genetic diversity was assessed for each genetic marker and the concatenated set using DnaSP software [29]. The total number of mutations (Eta), polymorphic sites (S), haplotypes (h), haplotype diversity (Hd), nucleotide diversity (pi) expressed as nucleotide differences per site between two sequences, the Theta index of the Eta by site (θ) and the theta index of the S were reported with the corresponding variations and standard deviations. Using the same software, a Tajima's *D* [36] evolutionary divergence test was performed, calculated as the difference between the average of differences among pairs of sequences and the number of segregating sites, and statistics of population structure such as Fst looking at population subdivision and genetic divergence patterns (Dxy). Finally, a Fu’s *F*_S_ test of selective neutrality was performed, based on the infinite sites model [37], in order to evaluate population growth at the departmental and national levels.

## Results

### Evaluation of phylogenetic relationships

**Fig. 1:**
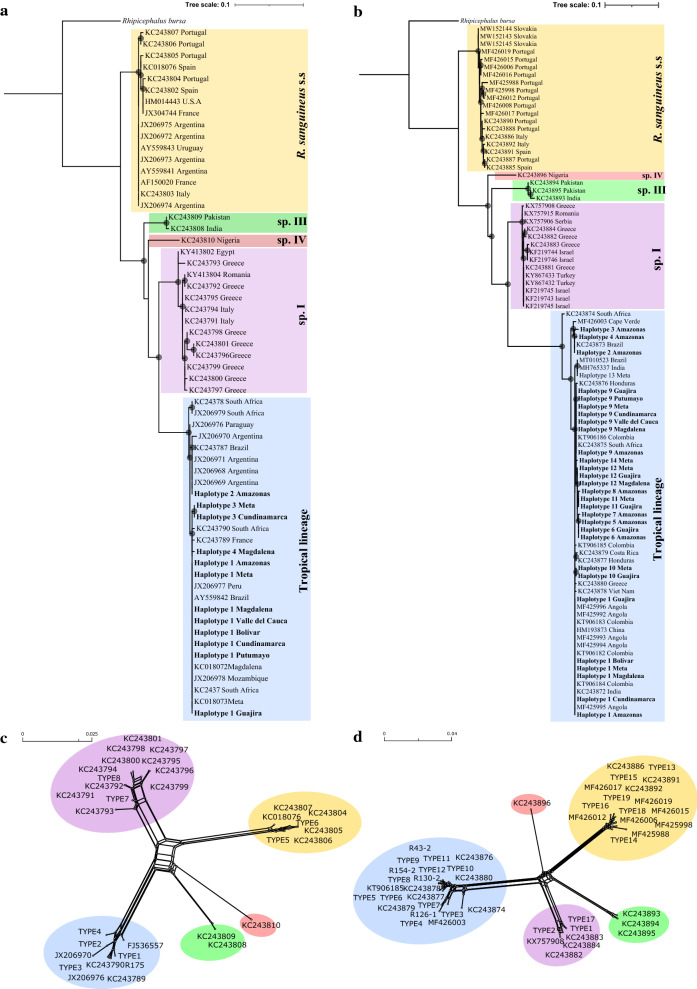
Trees and phylogenetic networks with available sequences. **a, c** 12S rDNA. **b, d** *COI*. *Rhipicephalus bursa* (GenBank: AF150053, KT313101) was used as outgroup. Bootstraps > 60% are shown. The sequences generated in this study are shown in bold

Alignment of haplotypes against available public sequences of mitochondrial markers (*COI* and 12S rDNA) showed similar topologies for both trees and phylogenetic networks (Fig. 1), in relationship with *R*. *sanguineus* s.s. (= temperate lineage), tropical lineage of *R*. *sanguineus* s.l., *Rhipicephalus* sp. I (also referred to as the southeastern Europe lineage [38]), *Rhipicephalus* sp. III and *Rhipicephalus* sp. IV [16]. All the haplotypes present in Colombia grouped under the tropical lineage of *R*. *sanguineus* s.l.

Using both markers, our samples showed a close relation with sequences identified from several other countries. The phylogenetic networks for both markers displayed the same groupings as their corresponding trees (Fig. 1c and d). Although usually exhibiting lineage divergence, cross-linking events were observed which could suggest the possibility of potential recombination or hybridization events among different *R*. *sanguineus* s.l. lineages. Lastly, the ITS2 marker analysis presented great differences in tree topology, lacking significant grouping and/or differentiation trends among lineages (Additional file 3: Figure S1).

### Haplotype networks

**Fig. 2:**
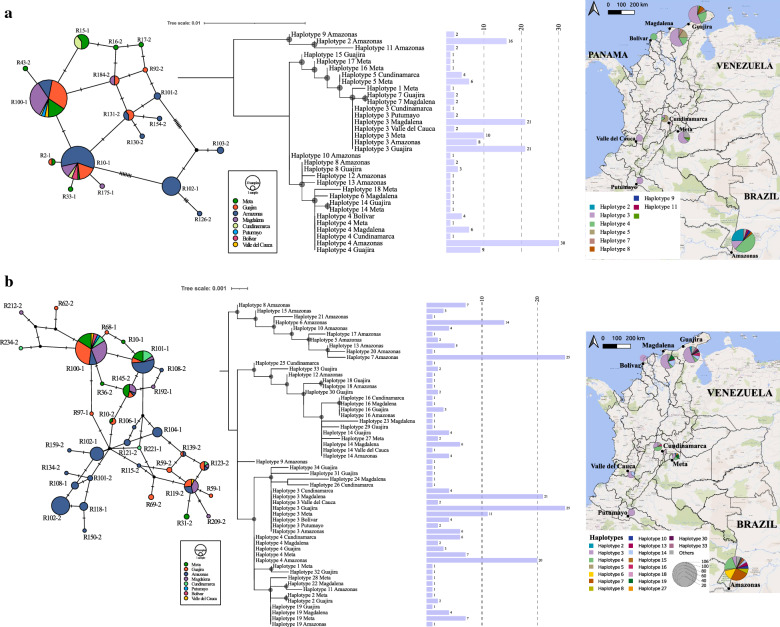
Haplotype networks, phylogenetic trees and haplotype distribution of the sequences generated in this study. **a** Concatenated mitochondrial markers: 12S rDNA and *COI*. **b** ITS2. Bootstraps > 60% are shown

Focusing on the population structure of *R*. *sanguineus* s.l. in Colombia, the haplotype network from the concatenated mitochondrial markers showed a total of 18 haplotypes (Fig. 2a), three of which were present exclusively in the Amazonas Department, separated by several sequence mutations from lineages detected elsewhere in the country. These sequences form a separated cluster in the phylogenetic tree, composed of haplotypes 2, 9 and 11, which represents 30.6% of the relative abundance of sequences in the Amazonas Department, and 18.8% of the total haplotypes countrywide. This same cluster was also identified in the phylogeny obtained from analysis of the ITS2 marker (Fig. 2b). For this genetic marker, the haplotype network revealed a total of 34 haplotypes, with the majority linked to a single sequence. Of these, 10 haplotypes grouped under the Amazonas cluster, which is equivalent to 29.4% of the total haplotypes identified, and 63.6% of the total sequences from the Amazonas Department. This clustering trend was also observed separately for the mitochondrial markers (Additional file 4: Figure S2).

Phylogenetic trees inclusive of publicly available sequences (Fig. 1a and b) revealed that the Amazon cluster grouped with samples from Brazil (GenBank: KC243787, KC243873), Argentina (GenBank: JX206968-JX206971) and Cape Town (GenBank: MF426003). The rest of the population failed to reveal any difference based on geographical area. Although two clusters were observed for the concatenated and ITS2 trees, no apparent geographical grouping by departments or natural regions could be identified (Fig. 2).

### Genetic diversity and population structure

Low levels of genetic diversity (pi and theta) were observed across all samples from Colombia, contrasting with the high number sequences available from around the world (Table 1). In terms of performance, 12S rDNA was the marker exhibiting the least genetic diversity (pi: 0,00,154; theta of Eta: 0,00,213), followed by *COI* (pi: 0,00,429; theta of Eta: 0,00,672) and finally ITS2 (pi: 0,00,393; theta of Eta: 0,00,289), where the concatenated mitochondrial markers showed a good intermediate representation between 12S rDNA and *COI* (Table 1).

**Table 1:** Analysis of genetic markers (individually and concatenated) using sequences generated in this study compared with the sequences available in GenBank

	12S rDNA		*COI*		ITS2		Concatenated M
	Colombia	GenBank	Colombia	GenBank	Colombia	GenBank	Colombia
Number of sequences	170	221	181	251	235	291	166
S	4	56	18	108	15	70	22
Eta	4	71	18	132	16	79	22
h	4	29	14	49	34	69	18
Hd	0.318	0.557	0.676	0.804	0.850	0.892	0.742
Var Hd	0.00188	0.00155	0.00058	0.00034	0.00026	0.00015	0.00052
sd Hd	0.043	0.039	0.024	0.018	0.016	0.0012	0.023
Pi	0.00154	0.02259	0.00429	0.03551	0.00393	0.00508	0.00336
Theta of eta	0.00213	0.03646	0.00672	0.04663	0.00289	0.01383	0.00488
Theta of S	0.00213	0.02876	0.00672	0.03815	0.00270	0.01226	0.00488
Var (no recombination)	0.0000013	0.0000505	0.0000045	0.0000744	0.0000008	0.000081	0.0000022
sd (no recombination)	0.00114	0.00710	0.00212	0.00862	0.00088	0.00285	0.00148
Var (recombination)	0.0000011	0.0000148	0.0000025	0.0000135	0.0000005	0.0000021	0.0000011
sd (recombination)	0.00106	0.00384	0.00158	0.00367	0.00070	0.00146	0.00104
Tajima's *D*	−0.50354	−1.14841	−0.96082	−0.62811	0.91559	−1.88183	−0.86051
Fu and Li's D*	−0.37596	−1.08061	−2.661119*	−1.24546	−0.80379	−5.56384**	−2.37026*
Fu and Li's F*	−0.49469	−1.33475	−2.36679*	−1.12848	−0.16609	−4.57960**	−2.13043

The total number of mutations (Eta), polymorphic sites (S), haplotypes (h), haplotype diversity (Hd), nucleotide diversity (pi) expressed as nucleotide differences per site between two sequences, the Theta index of the Eta by site (θ) and the theta index of the S were reported with the corresponding variations and standard deviations

*P < 0.05; ** *P* < 0.01

High levels of haplotype diversity were observed for most markers, with values ranging between 0.318 and 0.850, suggesting a recent population expansion. This hypothesis was supported by obtaining significant Fu and Li's D* and/or Fu and Li's F* values for one marker. *COI* with −2.661119 and −2.36679, respectively, proved significant for both statistical values, in addition to the concatenated sequences (*P* < 0.05), with a Fu and Li’s D* value of − 2.37026. In contrast, 12S rDNA and ITS2 failed to reveal significance for either statistical value. Tajima'D values were also very low (between −0.50354 and −0.96082), except in the ITS2 marker (0.9155), showing no statistical significance for any marker or the concatenated sequences.

**Fig. 3:**
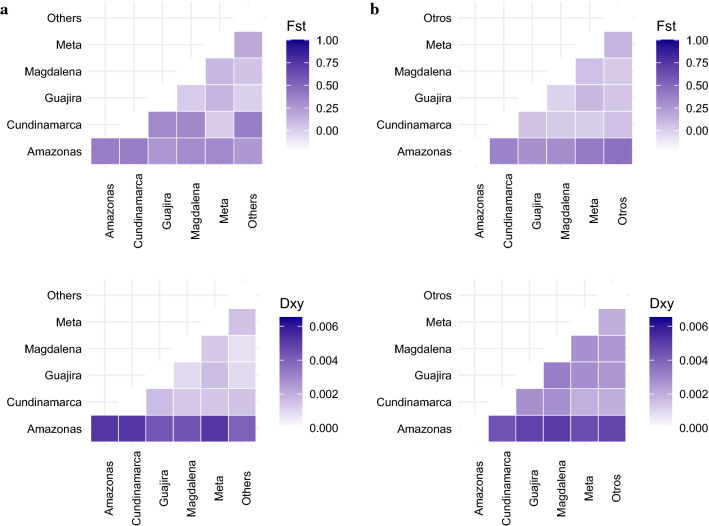
Fst and Dxy by department. **a** Concatenated mitochondrial markers: 12S rDNA and *COI*. **b** ITS

Finally, analysis of genetic diversity indexes by regions showed moderate values of Fst and Dxy in sequences from Amazonas (0.45049–0.31095; 0.00653–0.00355, respectively), a trend that was consistent for all markers evaluated. The Fu/Li and Tajima's *D* values were determined in these sequences but failed to show significance (Additional file 5: Table S4). However, low values of genetic structure for departments other than Amazonas were recorded (Fig. 3, Additional file 6: Figure S3). The Cundinamarca Department presented the highest Fst (0.57143, 0.54506 and 0.57143) values for 12S rDNA, whereas the Meta Department showed very low (0.01955) to moderate values when compared to the rest of the departments (0.33–0.36). Even though some clustering was observed for Meta and Cundinamarca in the 12S rDNA phylogenetic tree (Additional file 4: Figure S2), there were only eight samples from Cundinamarca, and this pattern was not reproducible for the *COI* or ITS2 markers. For example, values of 0.0519, 0.0265, 0.0093 and 0.06308 were observed in Cundinamarca for ITS2. These structure values were in part reflected in the concatenated set (0.10–0.38), but there was no clear clustering for these departments in the corresponding tree. The rest of the departments showed Fst values ranging from 0 to 0.10, and Dxy between 0 and 0.003, with some negative values of Fst in *COI* for Cundinamarca and Guajira.

## Discussion

Despite the wide distribution of *R*. *sanguineus* s.l. [4] and its relevance as a vector for a wide range of human and animal pathogens [5–7], little is known about the population genetics and diversity of this tick species in some tropical countries, including Colombia. Herein, we surveyed *R*. *sanguineus* s.l. from across different regions of Colombia in order to elucidate the phylogeographical landscape of this tick species through diverse regions and its potential influence on the observed genetic variation. Results obtained from previous studies have revealed the tropical lineage as the only clade currently circulating in Colombia [39]. In general, this observed genetic uniformity is thought to be associated with the well-known geographical restriction reported for *R*. *sanguineus* s.l. populations, as a consequence of their recognized limitations to adapt to different environmental conditions [20]. Intriguingly, previous observations reporting on the identification of a single tick grouping within *R*. *sanguineus* s.s. from Colombia [14] suggests that co-circulation of lineages may be taking place. However, a broader sampling throughout all regions is necessary to ascertain whether *R*. *sanguineus* s.s. is actually established in any geographical region of Colombia. In addition, other factors influencing the biology of this species need to be looked at, including (i) the range of host species, particularly wild synanthropic animals known to serve as hosts for these ectoparasites [4]; (ii) a broader geographical scale, in order to assess environmental heterogeneity; and, (iii) detailed studies at a micro-geographical scale focusing in fringe areas of the Colombian-Brazilian Amazon where co-circulation of *R*. *sanguineus* s.s. and tropical lineage have been reported [14]. Assessing these aspects along with finer aspects of host specificity, mobility and dispersal is of utmost importance to evaluate the influence of these factors on the genetic structure of *R*. *sanguineus* s.l. populations.

Our phylogenetic network analysis using all available sequences (Fig. 1) revealed some degree of cross-linking suggesting the possibility of hybridization amongst different lineages as proposed by other authors. For instance, Dantas-Torres and collaborators have previously reported on the viability of hybrids between *R*. *sanguineus* s.l. lineages [17], and their possible occurrence under natural conditions has also been suggested [38]. Given that multiple studies have documented hybridization events between different tick species [17] and that results from our phylogenetic network demonstrate divergent lineages among them (Fig. 1), it is possible that these may well represent different species as reported by previous works [16]. Still, future studies determining biological crosses are needed to further validate these findings.

When assessing levels of genetic variation in our study, we found that the ITS2 failed to finely resolve *R*. *sanguineus* s.l. lineages/species, grouping all the collected ticks into a single cluster. In fact, low resolution for species-level identification has been previously documented for *R*. *turanicus* [39], highlighting the potential pitfalls in informing not only for species but also at a genus level when interrogating this region [22]. In this sense, we strongly advise against the use of this individual marker for future phylogenetic studies between lineages.

Deciding on the most appropriate marker for tick-specific genetic population studies is a difficult task. However, mitochondrial genes have become the preferred standard given that their mutation rate enables resolution to a species level, proving highly informative for the purpose of phylogenetic and taxonomic studies. In our study, the use of concatenated mitochondrial markers allowed us to reconstruct phylogenetic relationships with higher confidence. We believe that following a combined approach that includes both the integration of new and further concatenation of mitochondrial markers will help achieve a higher resolution than using single gene-based molecular markers. Moreover, the inclusion of the analysis of mitochondrial genomes would provide better scrutiny of deep-level relationships, allowing us to reconcile phylogenetic controversies in tick taxonomy, particularly in the phylogenetic diversification of *R*. *sanguineus* s.l.

The present study provided new insights into the phylogenetic relationships and structuring of *R*. *sanguineus* s.l. in Colombia. We found a high diversity of haplotypes along with low nucleotide diversity for all markers, a pattern known to define the genetic signature of populations that had undergone geographical expansion followed by bottlenecks [40]. Such demographic expansion generates an excess of haplotypes and cumulative mutations [40], as evidenced from our haplotype networks (Fig. 2). The occurrence of this antecedent population expansion was confirmed after obtaining significant negative Fu's *F*_S_ (excess number of alleles) for most of the markers assessed (Table 1). Despite this, the obtained Tajima’s *D* values proved not to be significant, ranging between 0.50354 and −0.86051. These ranges graded lower than those obtained for other tick population expansion studies, such as *Amblyomma americanum*, where values fluctuating between −0.514 and −2.158 clearly signal an expansion after a recent bottleneck [41]. Though Fu's *F*_S_ statistics are more powerful in detecting demographic changes, they can also be affected by recombination [42]. Assuming the absence of a recombination event, further studies should be conducted following previous recommendations in order to corroborate the population expansion supported by both differentiation statistics and, in the near future, with more information, corroborate our hypothesis.

Because ticks exhibit a limited range for self-dispersion, genetic flow in these ectoparasites is known to be highly influenced by host mobility, thus impacting levels of population structure [43]. Therefore, species such as *A*. *americanum*, which infest high-mobility hosts, lack genetic structuring, as opposed to species such as *Amblyomma dissimile*, which infest low-mobility hosts, hence displaying high levels of genetic structuring [43]. In this study, we demonstrated low levels of genetic structure accompanied by high population expansion in most departments (Fig. 3). In fact, the low values obtained for the measures of genetic differentiation Fst and Dxy for *R*. *sanguineus* s.l. in our work appear to correlate with the high mobility of its main host, the domestic dog, which plays an important role in promoting tick dispersal through human transport across the country. Moreover, other hosts prone to tick infestation such as cattle and horses may contribute to dispersal between regions, as described in previous studies [39]. Indeed, the high mobility of cattle and other animals through different geographical areas, driven by guerrilla movements in Colombia during the last decades, might be fuelling the further spread and establishment of ticks in other areas via human-mediated transport.

In contrast, the Amazonas Department (Colombian Amazon Basin) reveals a different scenario, depicting moderate levels of structuring which could be explained based on its geographical remoteness and isolation and limited human and host mobility. A similar pattern of structuring has been documented for the tick *Dermacentor variabilis*, which also employs dogs as main hosts [43, 44]. Interestingly, this species shows high differentiation through isolated areas of the western USA compared to populations from Canada and central and eastern USA [44]. While it is possible that the observed moderate level of structuring observed in the Amazon is a consequence of geographical separation, other biological factors such as tick behavioural traits and host specificity, amongst others, may also influence gene flow levels and population structure, as demonstrated in some *Rhipicephalus* species [43]. Future studies are warranted to further investigate the various drivers shaping genetic variation and population structure in the Amazon region.

Information provided from population genetic structure studies is key to deciphering the many intricate aspects of host-vector-pathogen systems. In fact, different population structures are known to modulate diverse aspects of disease dynamics [45]. The low structure for *R*. *sanguineus* s.l. evidenced in this study could have important repercussions for pathogen transmission, considering that vertical transmission of pathogens such as *Phlebovirus* and *Coxiella* has been reported in ticks [46, 47]. Potential ongoing changes in flow and genetic variation in *R*. *sanguineus* population across different regions in Colombia could possibly lead to an increase in diversity of tick-borne pathogens. Understanding the genetic structure of these ticks serves as a proxy indicator to infer the potential spread of these pathogens. Because genetic differences among different tick lineages may influence the ability to transmit different pathogens, further studies would be very informative to determine the wide variety of pathogens that this species transmits both country-wide and in the Amazon region. Correlating patterns of tick genetic variation with pathogen profiling will provide the most valuable insights into the epidemiology of tick-borne diseases caused by pathogens transmitted by *R*. *sanguineus* s.l. in Colombia.

## Conclusions

This study is the first to characterize the genetic structure of *R*. *sanguineus* s.l. in Colombia. Although a wide range of departments and varied ecosystems were surveyed, our results revealed the exclusive circulation of the tropical lineage all across the country. However, circulation of other lineages cannot be ruled out, particularly in light of the evidence for genetic admixture observed in this study. Our study also found signs of a recent demographic expansion as supported by normality tests. No strong signs of genetic structuring were found, except for the Amazon region, highlighting the importance of future studies in order to elucidate genetic connectivity across different tick populations. The domestic dog appears to play the most important role in dispersal and thus in determining gene flow amongst *R*. *sanguineus* s.l. nationwide, except for the most isolated regions like the Amazon, where most of the bioecological features of this tick have yet to be deciphered. This study represents the first detailed approach for characterizing the population structure and geographical distribution of this vector in Colombia and its possible implications in disease transmission. Tick-associated pathogen profiling, potential circulation of other lineages and vector competency are intriguing questions to be addressed in future studies of *R*. *sanguineus* s.l. in Colombia.

## Supplementary Information

**Additional file 1:Table S1.** Information on the ticks collected in this study.

**Additional file 2:Table S2.** Primers used for amplification by PCR in 12S rDNA, COI, and ITS2. **Table S3**. Amplification conditions for each marker used.

**Additional file 3:Figure S1.** Phylogenetic tree with sequences available for the ITS2 marker. *Rhipicephalus bursa* (GenBank: KM986320) was used as outgroup. Bootstraps >60% are shown. Sequences generated in this study are in bold.

**Additional file 4:Figure S2.** Haplotype networks, phylogenetic trees, and haplotype distribution of the sequences generated in this study. a 12S rDNA. b COI. *Rhipicephalus leporis* (GenBank: FJ536557, KX757911) was used as outgroup. Bootstraps >60% are shown.

**Additional file 5:Table S4.** FU’s tests in the Amazonas Department and the phylogenetically separated population by the mitochondrial markers and their concatenated set (Concatenated M).

**Additional file 6:Figure S3.** Genetic structure values Fst and Dxy. a 12S rDNA. b COI.

## Data Availability

The datasets used and/or analysed during the current study are available in the supplementary files. The sequences were deposited in GenBank (MZ452706-MZ467616).

## Abbreviations

s.l.: Sensu Lato
*COI*: Cytochrome c oxidase I
ITS2: Internal transcribed spacer 2
