# Supplementary material for: Genetic diversity and population structure of Rhipicephalus sanguineus sensu lato across different regions of Colombia
Source: Parasit Vectors. 2021 Aug 23;14:424. doi: 10.1186/s13071-021-04898-w (PMC8383428; doi:10.1186/s13071-021-04898-w)
Supplement: Supplementary file 1 — Additional file 1:Table S1. Information on the ticks collected in this study. [file 13071_2021_4898_MOESM1_ESM.docx]

**Additional file 1: Table S1.** Information of the ticks collected in this study.

| **ID** | **Sex** | **Latitude** | **Longitude** | **Natural region** | **Depatment** | **City/town** | **Mean anual temperature ºC** |
| --- | --- | --- | --- | --- | --- | --- | --- |
| RS001-1 | Male | 0.500556 | -76.49889 | Amazonas | Putumayo | Pto Asis | 24.6 |
| RS001-2 | Male | 0.500556 | -76.49889 | Amazonas | Putumayo | Pto Asis | 24.6 |
| RS002-2 | Male | 4.3364601 | -74.36378 | Andes | Cundinamarca | Fusagasuga | 18.6 |
| RS002-4 | Male | 4.3364601 | -74.36378 | Andes | Cundinamarca | Fusagasuga | 18.6 |
| RS004-1 | Female | 4.3364601 | -74.36378 | Andes | Cundinamarca | Fusagasuga | 18.6 |
| RS005-1 | Female | 10.423611 | -75.52528 | Caribe | Bolivar | Cartagena | 27.2 |
| RS005-2 | Female | 10.423611 | -75.52528 | Caribe | Bolivar | Cartagena | 27.2 |
| RS006-1 | Female | 10.423611 | -75.52528 | Caribe | Bolivar | Cartagena | 27.2 |
| RS006-2 | Female | 10.423611 | -75.52528 | Caribe | Bolivar | Cartagena | 27.2 |
| RS007-1 | Male | 3.44 | -76.51972 | Pacifico | Valle del Cauca | Cali | 20.5 |
| RS008-1 | Male | 3.44 | -76.51972 | Pacifico | Valle del Cauca | Cali | 20.5 |
| RS008-2 | Male | 3.44 | -76.51972 | Pacifico | Valle del Cauca | Cali | 20.5 |
| RS011-1 | Female | 4.3364601 | -74.36378 | Andes | Cundinamarca | Fusagasuga | 18.6 |
| RS014-1 | Female | 4.3364601 | -74.36378 | Andes | Cundinamarca | Fusagasuga | 18.6 |
| RS017-8 | Male | 4.3364601 | -74.36378 | Andes | Cundinamarca | Fusagasuga | 18.6 |
| RS020-1 | Female | 4.1425 | -73.62944 | Orinoquia | Meta | Villavicencio | 24.0 |
| RS020-6 | Female | 4.1425 | -73.62944 | Orinoquia | Meta | Villavicencio | 24.0 |
| RS020-7 | Female | 4.1425 | -73.62944 | Orinoquia | Meta | Villavicencio | 24.0 |
| RS021-2 | Male | 4.1425 | -73.62944 | Orinoquia | Meta | Villavicencio | 24.0 |
| RS022-1 | Male | 4.1425 | -73.62944 | Orinoquia | Meta | Villavicencio | 24.0 |
| RS022-2 | Female | 4.1425 | -73.62944 | Orinoquia | Meta | Villavicencio | 24.0 |
| RS022-3 | Female | 4.1425 | -73.62944 | Orinoquia | Meta | Villavicencio | 24.0 |
| RS022-4 | Female | 4.1425 | -73.62944 | Orinoquia | Meta | Villavicencio | 24.0 |
| RS022-5 | Female | 4.1425 | -73.62944 | Orinoquia | Meta | Villavicencio | 24.0 |
| RS022-6 | Female | 4.1425 | -73.62944 | Orinoquia | Meta | Villavicencio | 24.0 |
| RS022-7 | Female | 4.1425 | -73.62944 | Orinoquia | Meta | Villavicencio | 24.0 |
| RS023-1 | Male | 4.1425 | -73.62944 | Orinoquia | Meta | Villavicencio | 24.0 |
| RS024-5 | Female | 4.1425 | -73.62944 | Orinoquia | Meta | Villavicencio | 24.0 |
| RS024-6 | Female | 4.1425 | -73.62944 | Orinoquia | Meta | Villavicencio | 24.0 |
| RS025-1 | Male | 4.1425 | -73.62944 | Orinoquia | Meta | Villavicencio | 24.0 |
| RS025-2 | Female | 4.1425 | -73.62944 | Orinoquia | Meta | Villavicencio | 24.0 |
| RS025-3 | Female | 4.1425 | -73.62944 | Orinoquia | Meta | Villavicencio | 24.0 |
| RS025-4 | Female | 4.1425 | -73.62944 | Orinoquia | Meta | Villavicencio | 24.0 |
| RS025-5 | Male | 4.1425 | -73.62944 | Orinoquia | Meta | Villavicencio | 24.0 |
| RS026-1 | Male | 4.1425 | -73.62944 | Orinoquia | Meta | Villavicencio | 24.0 |
| RS026-2 | Female | 4.1425 | -73.62944 | Orinoquia | Meta | Villavicencio | 24.0 |
| RS026-3 | Female | 4.1425 | -73.62944 | Orinoquia | Meta | Villavicencio | 24.0 |
| RS026-5 | Female | 4.1425 | -73.62944 | Orinoquia | Meta | Villavicencio | 24.0 |
| RS028-1 | Male | 11.544167 | -72.90694 | Caribe | Guajira | Riohacha | 27.2 |
| RS028-10 | Female | 11.544167 | -72.90694 | Caribe | Guajira | Riohacha | 27.2 |
| RS028-11 | Female | 11.544167 | -72.90694 | Caribe | Guajira | Riohacha | 27.2 |
| RS028-3 | Male | 11.544167 | -72.90694 | Caribe | Guajira | Riohacha | 27.2 |
| RS028-4 | Male | 11.544167 | -72.90694 | Caribe | Guajira | Riohacha | 27.2 |
| RS028-6 | Male | 11.544167 | -72.90694 | Caribe | Guajira | Riohacha | 27.2 |
| RS028-9 | Female | 11.544167 | -72.90694 | Caribe | Guajira | Riohacha | 27.2 |
| RS029-1 | Male | 11.544167 | -72.90694 | Caribe | Guajira | Riohacha | 27.2 |
| RS029-10 | Female | 11.544167 | -72.90694 | Caribe | Guajira | Riohacha | 27.2 |
| RS029-2 | Male | 11.544167 | -72.90694 | Caribe | Guajira | Riohacha | 27.2 |
| RS029-3 | Male | 11.544167 | -72.90694 | Caribe | Guajira | Riohacha | 27.2 |
| RS029-4 | Male | 11.544167 | -72.90694 | Caribe | Guajira | Riohacha | 27.2 |
| RS029-5 | Male | 11.544167 | -72.90694 | Caribe | Guajira | Riohacha | 27.2 |
| RS029-6 | Male | 11.544167 | -72.90694 | Caribe | Guajira | Riohacha | 27.2 |
| RS029-7 | Male | 11.544167 | -72.90694 | Caribe | Guajira | Riohacha | 27.2 |
| RS029-8 | Female | 11.544167 | -72.90694 | Caribe | Guajira | Riohacha | 27.2 |
| RS029-9 | Female | 11.544167 | -72.90694 | Caribe | Guajira | Riohacha | 27.2 |
| RS030-2 | Male | 11.544167 | -72.90694 | Caribe | Guajira | Riohacha | 27.2 |
| RS030-8 | Male | 11.544167 | -72.90694 | Caribe | Guajira | Riohacha | 27.2 |
| RS030-9 | Male | 11.544167 | -72.90694 | Caribe | Guajira | Riohacha | 27.2 |
| RS031-1 | Male | 11.544167 | -72.90694 | Caribe | Guajira | Riohacha | 27.2 |
| RS031-3 | Female | 11.544167 | -72.90694 | Caribe | Guajira | Riohacha | 27.2 |
| RS031-5 | Female | 11.544167 | -72.90694 | Caribe | Guajira | Riohacha | 27.2 |
| RS032-2 | Female | 11.544167 | -72.90694 | Caribe | Guajira | Riohacha | 27.2 |
| RS032-3 | Female | 11.544167 | -72.90694 | Caribe | Guajira | Riohacha | 27.2 |
| RS032-4 | Female | 11.544167 | -72.90694 | Caribe | Guajira | Riohacha | 27.2 |
| RS033-1 | Female | 11.544167 | -72.90694 | Caribe | Guajira | Riohacha | 27.2 |
| RS033-1 | Male | 11.544167 | -72.90694 | Caribe | Guajira | Riohacha | 27.2 |
| RS033-2 | Female | 11.544167 | -72.90694 | Caribe | Guajira | Riohacha | 27.2 |
| RS033-3 | Female | 11.544167 | -72.90694 | Caribe | Guajira | Riohacha | 27.2 |
| RS033-4 | Female | 11.544167 | -72.90694 | Caribe | Guajira | Riohacha | 27.2 |
| RS034-1 | Male | 11.544167 | -72.90694 | Caribe | Guajira | Riohacha | 27.2 |
| RS034-1 | Female | 11.544167 | -72.90694 | Caribe | Guajira | Riohacha | 27.2 |
| RS034-2 | Female | 11.544167 | -72.90694 | Caribe | Guajira | Riohacha | 27.2 |
| RS034-2 | Male | 11.544167 | -72.90694 | Caribe | Guajira | Riohacha | 27.2 |
| RS034-3 | Female | 11.544167 | -72.90694 | Caribe | Guajira | Riohacha | 27.2 |
| RS034-3 | Male | 11.544167 | -72.90694 | Caribe | Guajira | Riohacha | 27.2 |
| RS034-4 | Female | 11.544167 | -72.90694 | Caribe | Guajira | Riohacha | 27.2 |
| RS035-1 | Female | -4.215 | -69.94111 | Amazonas | Amazonas | Leticia | 25.5 |
| RS035-2 | Female | -4.215 | -69.94111 | Amazonas | Amazonas | Leticia | 25.5 |
| RS035-3 | Female | -4.215 | -69.94111 | Amazonas | Amazonas | Leticia | 25.5 |
| RS035-4 | Female | -4.215 | -69.94111 | Amazonas | Amazonas | Leticia | 25.5 |
| RS035-5 | Female | -4.215 | -69.94111 | Amazonas | Amazonas | Leticia | 25.5 |
| RS035-6 | Female | -4.215 | -69.94111 | Amazonas | Amazonas | Leticia | 25.5 |
| RS035-8 | Female | -4.215 | -69.94111 | Amazonas | Amazonas | Leticia | 25.5 |
| RS035-9 | Female | -4.215 | -69.94111 | Amazonas | Amazonas | Leticia | 25.5 |
| RS036-1 | Female | -4.215 | -69.94111 | Amazonas | Amazonas | Leticia | 25.5 |
| RS036-10 | Female | -4.215 | -69.94111 | Amazonas | Amazonas | Leticia | 25.5 |
| RS036-11 | Female | -4.215 | -69.94111 | Amazonas | Amazonas | Leticia | 25.5 |
| RS036-12 | Female | -4.215 | -69.94111 | Amazonas | Amazonas | Leticia | 25.5 |
| RS036-2 | Female | -4.215 | -69.94111 | Amazonas | Amazonas | Leticia | 25.5 |
| RS036-3 | Female | -4.215 | -69.94111 | Amazonas | Amazonas | Leticia | 25.5 |
| RS036-4 | Female | -4.215 | -69.94111 | Amazonas | Amazonas | Leticia | 25.5 |
| RS036-5 | Female | -4.215 | -69.94111 | Amazonas | Amazonas | Leticia | 25.5 |
| RS036-6 | Female | -4.215 | -69.94111 | Amazonas | Amazonas | Leticia | 25.5 |
| RS036-7 | Female | -4.215 | -69.94111 | Amazonas | Amazonas | Leticia | 25.5 |
| RS036-8 | Female | -4.215 | -69.94111 | Amazonas | Amazonas | Leticia | 25.5 |
| RS036-9 | Female | -4.215 | -69.94111 | Amazonas | Amazonas | Leticia | 25.5 |
| RS037-1 | Male | -4.215 | -69.94111 | Amazonas | Amazonas | Leticia | 25.5 |
| RS037-1 | Female | -4.215 | -69.94111 | Amazonas | Amazonas | Leticia | 25.5 |
| RS037-2 | Female | -4.215 | -69.94111 | Amazonas | Amazonas | Leticia | 25.5 |
| RS037-3 | Female | -4.215 | -69.94111 | Amazonas | Amazonas | Leticia | 25.5 |
| RS037-4 | Female | -4.215 | -69.94111 | Amazonas | Amazonas | Leticia | 25.5 |
| RS038-1 | Male | -4.215 | -69.94111 | Amazonas | Amazonas | Leticia | 25.5 |
| RS038-1 | Female | -4.215 | -69.94111 | Amazonas | Amazonas | Leticia | 25.5 |
| RS038-2 | Female | -4.215 | -69.94111 | Amazonas | Amazonas | Leticia | 25.5 |
| RS038-3 | Male | -4.215 | -69.94111 | Amazonas | Amazonas | Leticia | 25.5 |
| RS038-3 | Female | -4.215 | -69.94111 | Amazonas | Amazonas | Leticia | 25.5 |
| RS038-4 | Male | -4.215 | -69.94111 | Amazonas | Amazonas | Leticia | 25.5 |
| RS038-4 | Female | -4.215 | -69.94111 | Amazonas | Amazonas | Leticia | 25.5 |
| RS038-5 | Male | -4.215 | -69.94111 | Amazonas | Amazonas | Leticia | 25.5 |
| RS038-5 | Female | -4.215 | -69.94111 | Amazonas | Amazonas | Leticia | 25.5 |
| RS038-6 | Male | -4.215 | -69.94111 | Amazonas | Amazonas | Leticia | 25.5 |
| RS038-6 | Female | -4.215 | -69.94111 | Amazonas | Amazonas | Leticia | 25.5 |
| RS038-7 | Male | -4.215 | -69.94111 | Amazonas | Amazonas | Leticia | 25.5 |
| RS038-7 | Female | -4.215 | -69.94111 | Amazonas | Amazonas | Leticia | 25.5 |
| RS039-1 | Male | -4.215 | -69.94111 | Amazonas | Amazonas | Leticia | 25.5 |
| RS039-1 | Female | -4.215 | -69.94111 | Amazonas | Amazonas | Leticia | 25.5 |
| RS039-2 | Male | -4.215 | -69.94111 | Amazonas | Amazonas | Leticia | 25.5 |
| RS039-2 | Female | -4.215 | -69.94111 | Amazonas | Amazonas | Leticia | 25.5 |
| RS039-3 | Female | -4.215 | -69.94111 | Amazonas | Amazonas | Leticia | 25.5 |
| RS039-4 | Female | -4.215 | -69.94111 | Amazonas | Amazonas | Leticia | 25.5 |
| RS039-5 | Female | -4.215 | -69.94111 | Amazonas | Amazonas | Leticia | 25.5 |
| RS039-6 | Female | -4.215 | -69.94111 | Amazonas | Amazonas | Leticia | 25.5 |
| RS040-1 | Female | -4.215 | -69.94111 | Amazonas | Amazonas | Leticia | 25.5 |
| RS040-1 | Male | -4.215 | -69.94111 | Amazonas | Amazonas | Leticia | 25.5 |
| RS040-2 | Female | -4.215 | -69.94111 | Amazonas | Amazonas | Leticia | 25.5 |
| RS040-3 | Female | -4.215 | -69.94111 | Amazonas | Amazonas | Leticia | 25.5 |
| RS041-1 | Male | -4.215 | -69.94111 | Amazonas | Amazonas | Leticia | 25.5 |
| RS041-1 | Female | -4.215 | -69.94111 | Amazonas | Amazonas | Leticia | 25.5 |
| RS041-2 | Male | -4.215 | -69.94111 | Amazonas | Amazonas | Leticia | 25.5 |
| RS041-2 | Female | -4.215 | -69.94111 | Amazonas | Amazonas | Leticia | 25.5 |
| RS042-1 | Male | -4.215 | -69.94111 | Amazonas | Amazonas | Leticia | 25.5 |
| RS042-1 | Female | -4.215 | -69.94111 | Amazonas | Amazonas | Leticia | 25.5 |
| RS042-2 | Female | -4.215 | -69.94111 | Amazonas | Amazonas | Leticia | 25.5 |
| RS043-1 | Female | -4.215 | -69.94111 | Amazonas | Amazonas | Leticia | 25.5 |
| RS043-2 | Male | -4.215 | -69.94111 | Amazonas | Amazonas | Leticia | 25.5 |
| RS044-1 | Female | 11.236111 | -74.20167 | Caribe | Magdalena | Santa Marta | 25.6 |
| RS044-2 | Female | 11.236111 | -74.20167 | Caribe | Magdalena | Santa Marta | 25.6 |
| RS045-2 | Female | 11.236111 | -74.20167 | Caribe | Magdalena | Santa Marta | 25.6 |
| RS046-1 | Male | 11.236111 | -74.20167 | Caribe | Magdalena | Santa Marta | 25.6 |
| RS046-3 | Female | 11.236111 | -74.20167 | Caribe | Magdalena | Santa Marta | 25.6 |
| RS046-4 | Female | 11.236111 | -74.20167 | Caribe | Magdalena | Santa Marta | 25.6 |
| RS047-1 | Male | 11.236111 | -74.20167 | Caribe | Magdalena | Santa Marta | 25.6 |
| RS047-2 | Female | 11.236111 | -74.20167 | Caribe | Magdalena | Santa Marta | 25.6 |
| RS048-1 | Female | 11.236111 | -74.20167 | Caribe | Magdalena | Santa Marta | 25.6 |
| RS048-1 | Male | 11.236111 | -74.20167 | Caribe | Magdalena | Santa Marta | 25.6 |
| RS048-3 | Female | 11.236111 | -74.20167 | Caribe | Magdalena | Santa Marta | 25.6 |
| RS048-4 | Female | 11.236111 | -74.20167 | Caribe | Magdalena | Santa Marta | 25.6 |
| RS049-1 | Female | 11.236111 | -74.20167 | Caribe | Magdalena | Santa Marta | 25.6 |
| RS049-1 | Male | 11.236111 | -74.20167 | Caribe | Magdalena | Santa Marta | 25.6 |
| RS049-2 | Male | 11.236111 | -74.20167 | Caribe | Magdalena | Santa Marta | 25.6 |
| RS049-5 | Male | 11.236111 | -74.20167 | Caribe | Magdalena | Santa Marta | 25.6 |
| RS050-1 | Male | 11.236111 | -74.20167 | Caribe | Magdalena | Santa Marta | 25.6 |
| RS050-1 | Female | 11.236111 | -74.20167 | Caribe | Magdalena | Santa Marta | 25.6 |
| RS051-1 | Male | 11.236111 | -74.20167 | Caribe | Magdalena | Santa Marta | 25.6 |
| RS051-2 | Female | 11.236111 | -74.20167 | Caribe | Magdalena | Santa Marta | 25.6 |
| RS051-2 | Male | 11.236111 | -74.20167 | Caribe | Magdalena | Santa Marta | 25.6 |
| RS051-3 | Female | 11.236111 | -74.20167 | Caribe | Magdalena | Santa Marta | 25.6 |
| RS051-3 | Male | 11.236111 | -74.20167 | Caribe | Magdalena | Santa Marta | 25.6 |
| RS051-4 | Female | 11.236111 | -74.20167 | Caribe | Magdalena | Santa Marta | 25.6 |
| RS051-4 | Male | 11.236111 | -74.20167 | Caribe | Magdalena | Santa Marta | 25.6 |
| RS052-1 | Male | 11.236111 | -74.20167 | Caribe | Magdalena | Santa Marta | 25.6 |
| RS052-1 | Female | 11.236111 | -74.20167 | Caribe | Magdalena | Santa Marta | 25.6 |
| RS052-2 | Female | 11.236111 | -74.20167 | Caribe | Magdalena | Santa Marta | 25.6 |
| RS053-1 | Male | 11.236111 | -74.20167 | Caribe | Magdalena | Santa Marta | 25.6 |
| RS053-1 | Female | 11.236111 | -74.20167 | Caribe | Magdalena | Santa Marta | 25.6 |
| RS054-1 | Female | 11.236111 | -74.20167 | Caribe | Magdalena | Santa Marta | 25.6 |
| RS055-1 | Male | 11.236111 | -74.20167 | Caribe | Magdalena | Santa Marta | 25.6 |
| RS055-2 | Female | 11.236111 | -74.20167 | Caribe | Magdalena | Santa Marta | 25.6 |
| RS060-1 | Female | 4.730556 | -74.26389 | Andes | Cundinamarca | Madrid | 15.2 |
| RS060-2 | Female | 4.730556 | -74.26389 | Andes | Cundinamarca | Madrid | 15.2 |
