# Supplementary material for: Genetic diversity and population structure of Rhipicephalus sanguineus sensu lato across different regions of Colombia
Source: Parasit Vectors. 2021 Aug 23;14:424. doi: 10.1186/s13071-021-04898-w (PMC8383428; doi:10.1186/s13071-021-04898-w)
Supplement: Supplementary file 2 — Additional file 2:Table S2. Primers used for amplification by PCR in 12S rDNA, COI, and ITS2. Table S3. Amplification conditions for each marker used. [file 13071_2021_4898_MOESM2_ESM.docx]

**Additional file 2: Table S2.** Primers used for amplification by PCR in 12S rDNA, COI and ITS2

| Genetic marker | Primer name | F/R | Sequence | Reference |
| --- | --- | --- | --- | --- |
| COI | HCO2064 | Forward | GGTGGGCTCATACAATAAATCC | [22] |
|  | HCO1215 | Reverse | GCCATTTTACCGCGATGA |  |
| 12S rDNA | T2A | Forward | AAACTAGGATTAGATACCCT | [25] |
|  | 12S-R | Reverse | CTATGTAACGACTTATCTTAATAAAGAGTG | [24] |
| ITS2 | ITS-F | Forward | ACATTGCGGCCTTGGGTCTT | [23] |
|  | ITS-R | Reverse | TCGCCTGATCTGAGGTCGAC |  |

**Additional file 2: Table S3.** Amplification conditions for each marker used

| Stage | ITS2-F/ITS2-R | | | COX1F/COX1R | | | 12SF/12SR | | |
| --- | --- | --- | --- | --- | --- | --- | --- | --- | --- |
|  | Temperature | Time | Cycles | Temperature | Time | Cycles | Temperature | Time | Cycles |
| Initial denaturation | 95 °C | 5 min | - | 94 °C | 5 min | - | 94 °C | 5 min | - |
| Denaturation | 95 °C | 30 s | 5 | 94 °C | 30 s | 5 | 94 °C | 30 s | 5 |
| Annealing | 58 °C | 30 s |  | 52 °C | 30 s |  | 48 °C | 30 s |  |
| Extension | 72 °C | 90 s |  | 68 °C | 60 s |  | 72 °C | 60 s |  |
| Denaturation | 94 °C | 30 s | 30 | 94 °C | 30 s | 5 | 94 °C | 30 s | 5 |
| Annealing | 56 °C | 30 s |  | 50 °C | 30 s |  | 50 °C | 30 s |  |
| Extension | 72 °C | 90 s |  | 68 °C | 60 s |  | 72 °C | 60 s |  |
| Denaturation |  |  |  | 94 °C | 30 s | 5 | 94 °C | 30 s | 5 |
| Annealing |  |  |  | 48 °C | 30 s |  | 52 °C | 30 s |  |
| Extension |  |  |  | 68 °C | 60 s |  | 72 °C | 60 s |  |
| Denaturation |  |  |  | 94 °C | 30 s | 25 | 94 °C | 30 s | 20 |
| Annealing |  |  |  | 46 °C | 30 s |  | 46 °C | 30 s |  |
| Extension |  |  |  | 68 °C | 60 s |  | 72 °C | 60 s |  |
| Final extension | 72 °C | 5 min | - | 68 °C | 5 min | - | 72 °C | 5 min | - |
