# Supplementary figures and images for: Genetic diversity and population structure of Rhipicephalus sanguineus sensu lato across different regions of Colombia
Source: Parasit Vectors. 2021 Aug 23;14:424. doi: 10.1186/s13071-021-04898-w (PMC8383428; doi:10.1186/s13071-021-04898-w)

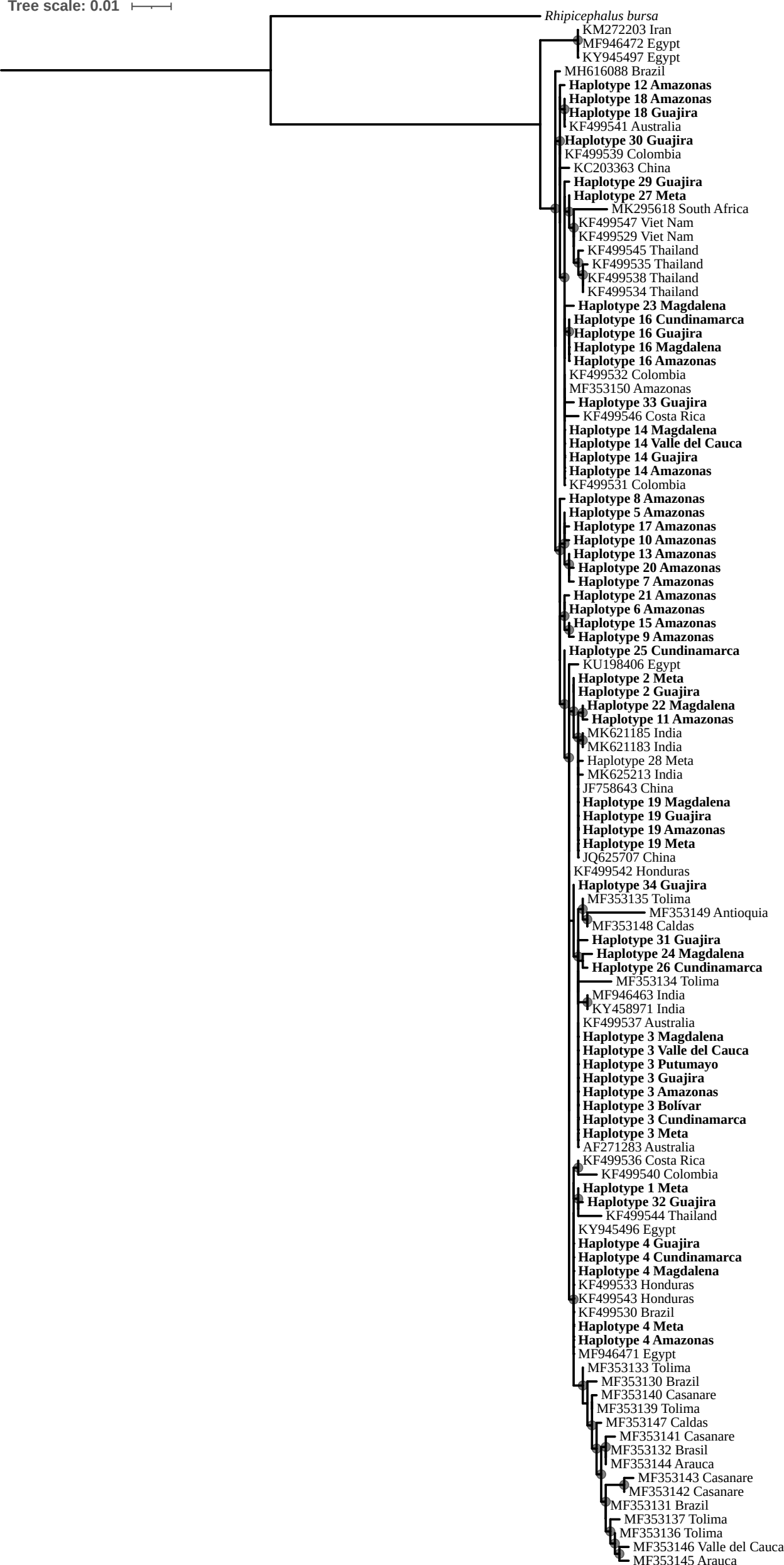

Supplement: Supplementary file 3 — Additional file 3:Figure S1. Phylogenetic tree with sequences available for the ITS2 marker. Rhipicephalus bursa (GenBank: KM986320) was used as outgroup. Bootstraps >60% are shown. Sequences generated in this study are in bold. [file 13071_2021_4898_MOESM3_ESM.pdf]

A

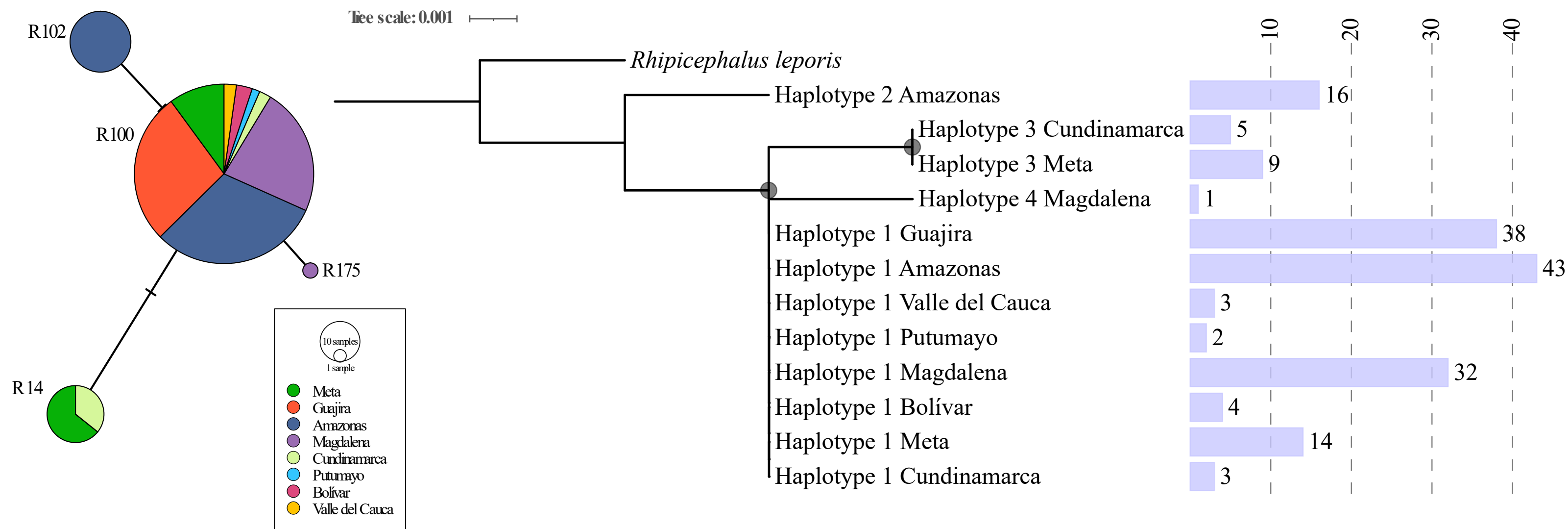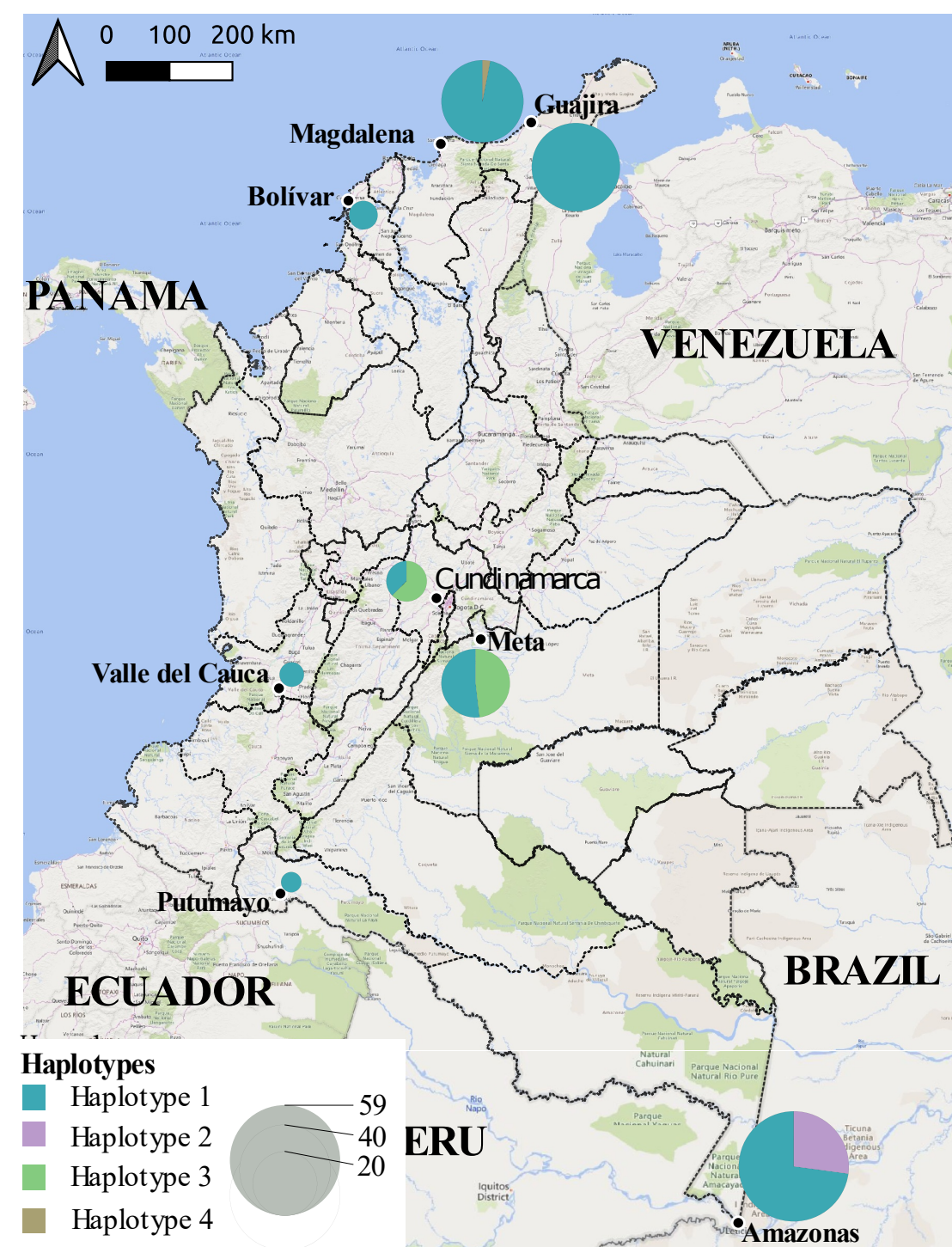

B

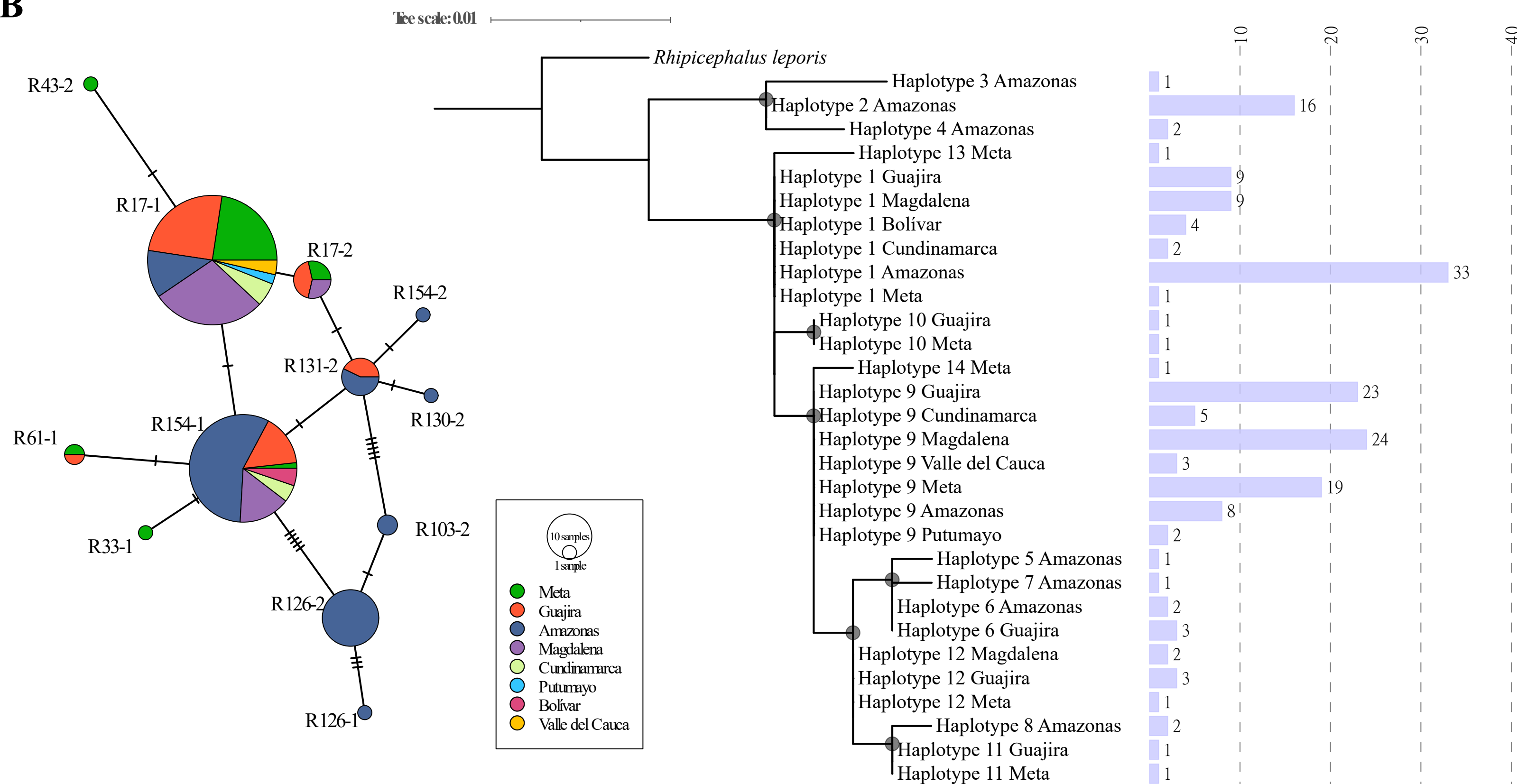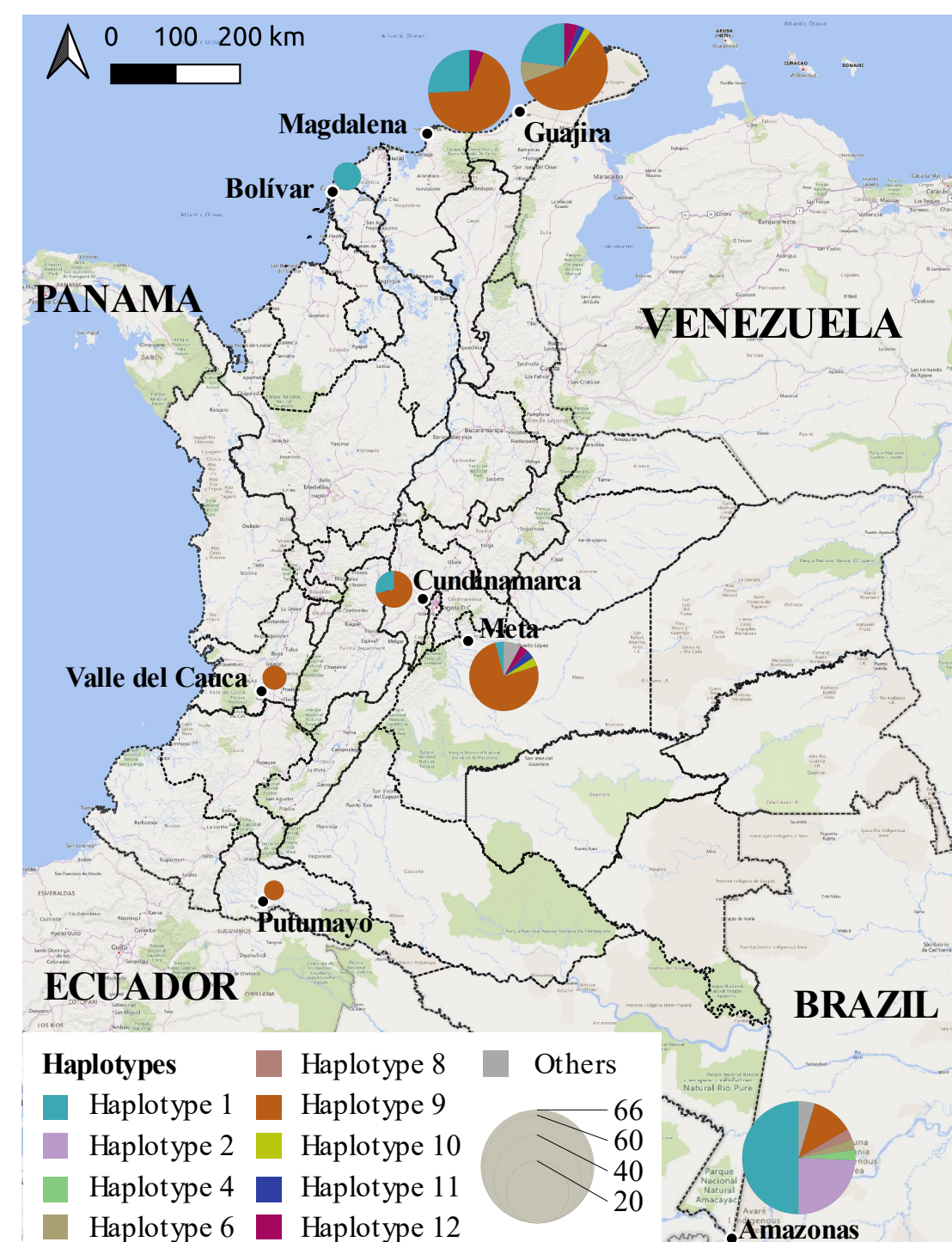

Supplement: Supplementary file 4 — Additional file 4:Figure S2. Haplotype networks, phylogenetic trees, and haplotype distribution of the sequences generated in this study. a 12S rDNA. b COI. Rhipicephalus leporis (GenBank: FJ536557, KX757911) was used as outgroup. Bootstraps >60% are shown. [file 13071_2021_4898_MOESM4_ESM.pdf]

**A**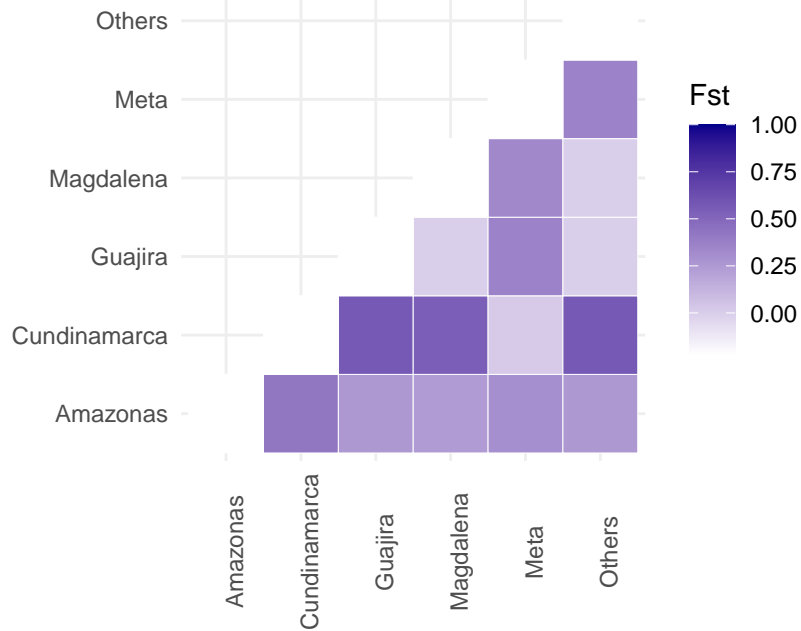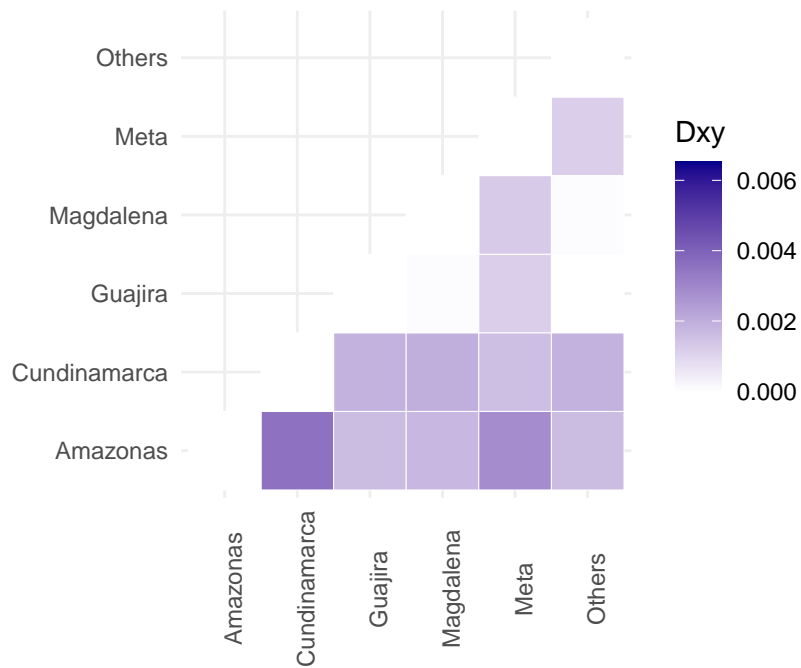**B**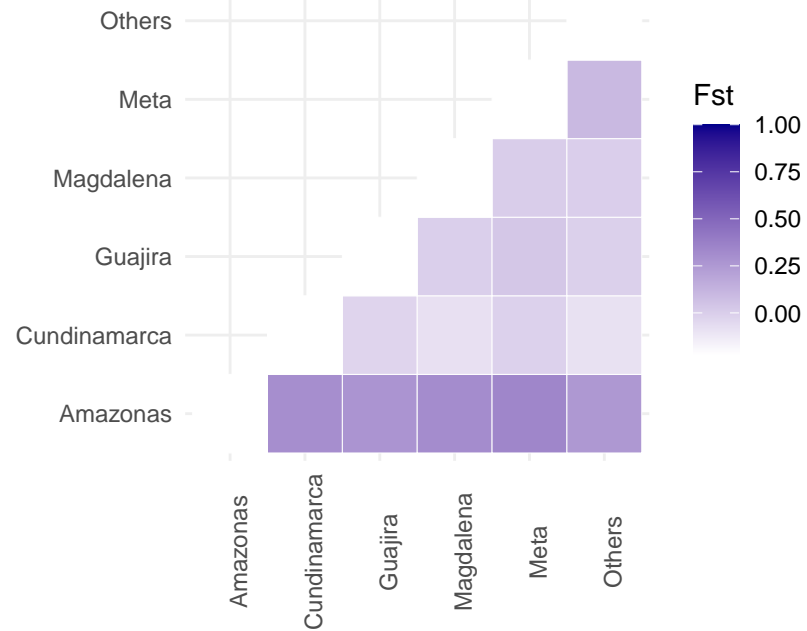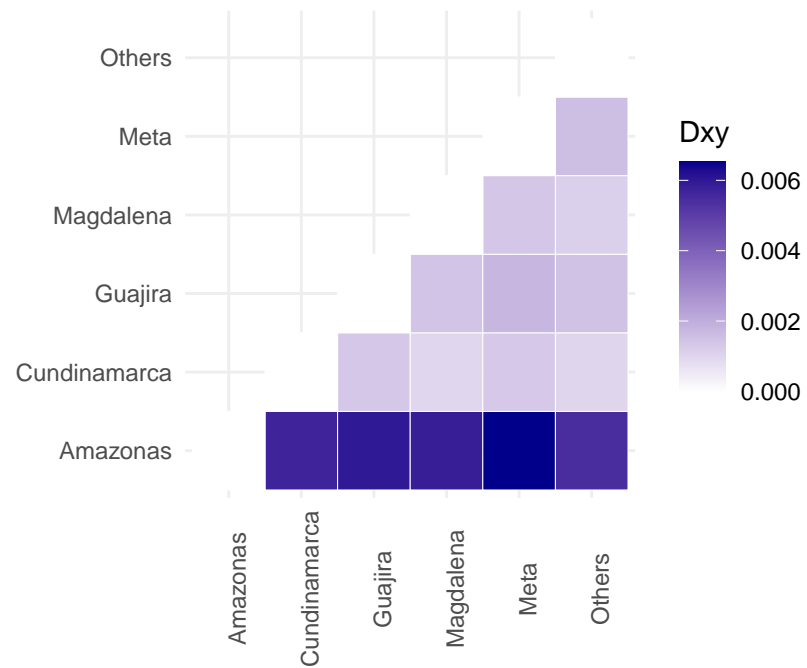

Supplement: Supplementary file 5 — Additional file 5:Table S4. FU’s tests in the Amazonas Department and the phylogenetically separated population by the mitochondrial markers and their concatenated set (Concatenated M). [file 13071_2021_4898_MOESM5_ESM.pdf]
