# Supplementary material for: Genetic diversity and population structure of Rhipicephalus sanguineus sensu lato across different regions of Colombia
Source: Parasit Vectors. 2021 Aug 23;14:424. doi: 10.1186/s13071-021-04898-w (PMC8383428; doi:10.1186/s13071-021-04898-w)
Supplement: Supplementary file 6 — Additional file 6:Figure S3. Genetic structure values Fst and Dxy. a 12S rDNA. b COI. [file 13071_2021_4898_MOESM6_ESM.docx]

**Additional file 6: Table S4.** FU’s tests in the department of Amazonas and in the phylogenetically separated population by the mitochondrial markers and their concatenate (ConcatenatedM).

| Population | Statistics | ConcatenatedM | COI | 12S rDNA |
| --- | --- | --- | --- | --- |
| Amazonas | Tajima´D | 0.66771 | 0.25261 | 1.48985 |
|  | Fu and Li's D* | -0.72292 | -1.04401 | 0.73098 |
|  | Fu and Li's F* | -0.28005 | -0.70322 | 1.11371 |
| Subset Amazonas | Tajima'D | -0.86051 | -1.55897 | -0.5035 |
|  | Fu and Li's D* | -1.17653 | -1.17653 | 0 |
|  | Fu and Li's F* | -1.47757 | -1.47757 | 0 |
